# Retraction: Elevated Expression Levels of PC3-Secreted Microprotein (PSMP) in Prostate Cancer Associated With Increased Xenograft Growth and Modification of Immune-Related Microenvironment

**DOI:** 10.3389/fonc.2020.636664

**Published:** 2020-12-23

**Authors:** Frontiers Editorial Office

Following publication, concerns were raised regarding the duplication of the data presented in panels B and C of Figure 7 (*Frontiers in Oncology*, August 2019) and in panels A and B of Figure 7 in one of the author’s previous publications (*J. Immunol*. 2014 Feb 15;192(4):1878-86. doi: 10.4049/jimmunol.1300758). The authors failed to provide a satisfactory explanation.

This retraction was approved by the Chief Editors of Frontiers in Oncology.

The authors disagree with the retraction.

